# Optical Absorption Properties of Sn- and Pd-doped ZnO: Comparative Analysis of Substitutional Metallic Impurities

**DOI:** 10.3390/ma18194613

**Published:** 2025-10-05

**Authors:** Vicente Cisternas, Pablo Díaz, Ulises Guevara, David Laroze, Eduardo Cisternas

**Affiliations:** 1 Departamento de Ciencias Físicas, Universidad de La Frontera, Temuco 4780000, Chile; vicente.cisternas@gmail.com (V.C.); pablo.diaz@ufrontera.cl (P.D.); 2 Vicerrectoría de Investigación y Postgrado, Universidad de La Serena, La Serena 1700000, Chile; ujguev@gmail.com; 3 Instituto de Alta Investigación, Universidad de Tarapacá, Arica 1000000, Chile; dlarozen@uta.cl

**Keywords:** DFT calculations, optical absorption properties, electronic properties

## Abstract

In this article, we present density functional theory (DFT) calculations for $Zn_{\left(1-x\right)}M_{x}O$, where M represents one of the following substitutional metallic impurities: Ga, Cd, Cu, Pd, Ag, In, or Sn. Our study is based on the wurtzite structure of pristine ZnO. We employ the Quantum Espresso package, using a fully unconstrained implementation of the generalized gradient approximation (GGA) with an additional U correction for exchange and correlation effects. We analyze the density of states, energy gaps, and absorption spectra for these doped systems, considering the limitations of a finite-size cell approximation. Rather than focusing on precise numerical values, we highlight the following two key aspects: the location of impurity-induced electronic states and the overall trends in optical properties across the eight systems, including pristine ZnO. Our results indicate that certain dopants introduce electronic levels within the band gap, which enhance optical absorption in the visible, near-infrared, and near-ultraviolet regions. For instance, Sn-doped ZnO shows a pronounced absorption peak at ∼2.5 eV, which is in the middle of the visible spectrum. In the case of Ag and Pd impurities, they lead to increased electromagnetic radiation absorption at the near ultra-violet spectrum. This represents a promising performance for efficient solar radiation absorption, both at the Earth’s surface and in outer space. Furthermore, Ga- and In-doped ZnO present bandgaps of ∼0.9 eV, promising an interesting performance in the near infrared region. These findings suggest potential applications in solar energy harvesting and selective sensors.

## 1. Introduction

Zinc oxide (ZnO) has attracted important scientific and technological interest. In addition to well-known applications in the rubber and concrete industries [1], we can mention its use in various fields such as gas sensors, lasers, and photocatalysts [2,3,4,5,6]. ZnO is a semiconductor that shows a direct band gap (∼3.4 eV), which has also been applied in UV photodetectors [7] and catalysts [8], as well as antibacterial agents due to its low cost and reduced toxicity [9]. Furthermore, ZnO has piezoelectric and pyroelectric properties, which can be used in piezoelectric sensors [10]. In addition, ZnO nanostructures have been extensively investigated for chemical and optical sensing applications, offering high sensitivity and selectivity due to their large surface-to-volume ratio [11]. ZnO is also widely explored as a transparent conducting oxide (TCO), often alloyed with Al (AZO) or combined with Ag multilayers to enhance conductivity and optical transmittance [12,13]. ZnO nanostructures have demonstrated a remarkable performance in photocatalytic degradation processes, which include doping strategies under visible light [14], the treatment of hazardous and non-biodegradable pharmaceuticals [15], and the design of S-scheme heterojunction photocatalysts [16].

ZnO has also been proposed for solar energy harvesting solutions [17,18,19], particularly in dye-sensitized solar cells [20]. Moreover, although ZnO presents a wide band gap, it has been observed that some substitutional impurities can reduce this value [21,22,23], enabling the absorption of solar radiation. For example, theoretical calculations show that Fe impurities [21] and Cr impurities [22] can increase the electromagnetic absorption capacity of ZnO in the visible-light region. This absorption shift toward visible light has been experimentally observed for ZnO doped with Cr, Fe, and Ni [23]. In the same way, a combined theory–experimental study on ${\mathrm{Zn}}_{1-x}{\mathrm{Cu}}_{x}O$ shows that this compound has better optical absorption properties than pure ZnO [24]. Also noteworthy is an experimental study on ZnO nanocrystals doped with rare earths that shows a red shift at the absorption edge of the optical absorption spectra [25]. Furthermore, recent work on hybrid nanoparticles (ZnO-Al_2_O_3_) has demonstrated an enhanced performance for photovoltaic thermal energy storage [26]. However, although electromagnetic radiation absorption in the visible spectra is especially relevant due to the maximum solar spectral irradiance at Earth’s surface, the near-infrared and ultraviolet absorption bands could also be of interest for solar energy harvesting in outer space, where solar cells tend to combine a broad absorption spectrum with flexibility, while keeping costs low [27].

Recent works show that the functionalization of ZnO by doping remains the subject of intense study. For example, doping ZnO with Eu^3+^ ions for use as a quantum dot (QD) material shows great potential for use in photonic devices, light-emitting diodes, and bioimaging [28]. Another example shows a successful result, in terms of crystal structure stability, when doping ZnO with Ni^2+^ and Co^2+^ ions, resulting in a band gap value ranging from 3.18 eV for ZnO to 3.20 eV for ZnO:Ni and 2.82 eV for ZnO:Co [29]. Along the same lines, Nb doping has been reported to improve photoconversion efficiency by downshifting the luminescence, thus broadening the absorption range of the material in photovoltaic applications. The synthesized samples show a bandgap of 3.35 eV, a transmittance of 46.54%, and an absorbance of 0.33 a.u. [30]. Furthermore, in a recent theoretical work, first-principles calculations were used to study the structural stability and optical responses of Mn- and Y-doped ZnO systems, highlighting the importance of further developing predictive design for targeted property optimization [31]. Intensive experimental and theoretical work demonstrates the potential of doping and advanced synthesis methods in engineering ZnO-based materials for next-generation energy and photonic technologies.

This work investigates the role of substitutional impurities in modifying the structural, electronic, and optical absorption properties of doped ZnO. To this end, we have selected as dopants those elements neighboring Zn in the periodic table (i.e., Cu, Ga, Pd, Ag, Cd, In, and Sn). These elements were chosen primarily because their atomic radii are comparable to that of Zn, minimizing lattice distortion. Furthermore, these impurities can act as either donors or acceptors, altering the electronic structure near the Fermi level and, consequently, the electromagnetic absorption properties of pure ZnO. Additionally, the use of substitutional impurities with radii similar to Zn could offer an energetically favorable synthesis route, which may facilitate experimental validation of our theoretical predictions.

To ensure clarity, the manuscript is organized as follows: Section 2 outlines the theoretical approach, including the system model and computational details. Section 3 presents and discusses the main results, including structural parameters, density of states, and optical absorption properties. Finally, Section 4 summarizes the key findings and conclusions of this study.

## 2. Model and Computational Details

We have focused on the wurtzite phase of the ZnO crystal structure because it is stable at room temperature and ambient pressure, both of which are required for practical applications. The wurtzite phase corresponds to two hexagonal close-packed sublattices of cations Zn^2+^ and anions O^2−^, showing the following lattice parameters: a=b=3.2475 and c=5.2075 [32].

**Figure 1:**
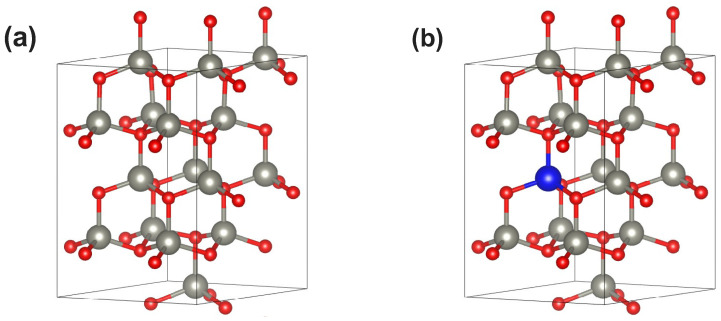
(**a**) Unit cell measuring 2 × 2 × 2 modeling pristine ${\mathrm{Zn}}_{m}O_{m}$ (m=16) in the wurzite phase. Zn(O) atoms appear represented in gray (red). (**b**) A substitutional metallic impurity (blue ball) leading to ${\mathrm{Zn}}_{m-1}{\mathrm{MO}}_{m}$. In particular, l=1 corresponds to an impurity concentration of 6.25%.

We have performed computational calculations employing the density functional theory (DFT) machinery. We have considered a supercell equivalent to 2×2×2 unit cells of ZnO, which corresponds to ${\mathrm{Zn}}_{m}O_{n}$ with m=n=16, i.e., N=32 atoms in the supercell (see Figure 1a). Periodic boundary conditions mimic the pristine bulk ZnO, while substitutional metallic impurities (M) lead to ${\mathrm{Zn}}_{m-l}M_{l}O_{m}$ (see Figure 1b). For l=1, the impurities concentration reaches 1/16=6.25%, which is a fixed parameter in what follows. This value provides a reasonable estimation of what can be achieved from an experimental perspective. In fact, higher concentrations could lead to undesirable impurity interactions, while lower and more realistic experimental concentrations could have only subtle effects on the physical properties of pristine ZnO. As stated before, the following substitutional elements M were considered: Cu, Ga, Pd, Ag, Cd, In, and Sn.

The structural parameters and the electronic structure were calculated employing the Quantum ESPRESSO distribution [33]. The core electrons were described by norm-conserving pseudo-potentials (NC-PPs) produced by the code Optimized Norm-Conserving Vanderbilt pseudopotentials [34]. For Cu, we included an NC-PP generated by Hartwigsen, Goedecker, and Hutter [35]. Exchange and correlation interactions were tackled under the LDA+U approximation [36,37] in the form of Perdew–Burke–Ernzerhof (PBE) [38]. In order to reproduce the experimental bandgap of the pristine matrix [39], the following Hubbard’s corrections were introduced: 5.0 eV for Zn and 10.0 eV for O. Such values were tuned on base those employed by Ma et al. [24], while they are also sustained by Ref. [40]. We have performed structural relaxation under a spin-polarized frame, with 1 meV as convergence criteria for the total energy. The first Brillouin zone of the supercell was sampled employing a Monkhorst–Pack mesh of 4 × 4 × 3, which ensures a correct convergence (see Figure S1 on Supplementary Materials). A plane-wave basis set with a 70 Ry energy cutoff for the wavefunctions (and 280 Ry for charge density) ensures an appropriate self-consistent calculation. However, the required energy cutoff for Cd and Cu impurities was 90 Ry and 140 Ry, respectively (meanwhile, 360 Ry and 560 Ry for charge density). The Broyden–Fletcher–Goldfarb–Shanno quasi-Newton algorithm [41] was performed for the ion dynamics, with 0.025 eV/Å as the force convergence criteria.

The optical properties of the compounds under study were obtained employing the epsilon.x code available in the Quantum ESPRESSO distribution [33].

## 3. Results and Discussion

### 3.1. Structures and Energies

The calculated lattice parameters for pristine ZnO show excellent agreement with both experimental data [32,42] and previous DFT results [43,44,45]. Furthermore, the introduction of substitutional impurities results in lattice constants that remain close to those of pristine ZnO, indicating that the structural stability of the material is preserved for each dopant (see Table 1).

Furthermore, magnetism does not play a significant role in the systems under study, except in the cases of palladium and copper. In fact, we can corroborate that thermal effects at room temperature (25 meV) are enough to demagnetize the spin-polarized configuration induced by a Pd impurity. This statement follows from the following definition of demagnetization energy per atom: $\Delta \epsilon_{p}=\left(E_{paramagn}-E_{spin-polar}\right)/N$, where $E_{paramagn}$ and $E_{spin-polar}$ correspond to the total energies in a paramagnetic and in a spin-polarized calculation, respectively (see the second last column of Table 1). In the case of Cu-doped ZnO, the magnetization per atom remains at room temperature, so it deserves to be analyzed separately, which is performed in the following subsection.

The band structure of pristine ZnO (wurzite phase) shows a quite good agreement with previously reported calculations (see Figure S2 on Supplementary Materials). Regarding the calculated energy bandgaps, the pristine ZnO reaches the well-known value of 3.4 eV, while the dopants notoriously modify this parameter. For example, In and Sn almost close the band gap of the pristine crystal, while Ga and Cd do not introduce an appreciable change.

The formation energy for ${\mathrm{Zn}}_{m-l}M_{l}O_{n}$ is defined as follows [46,47,48]:

(1) $$E_{\mathrm{Formation}}\left[{\mathrm{Zn}}_{m-l}M_{l}O_{n}\right]=E_{\mathrm{Total}}\left[{\mathrm{Zn}}_{m-l}M_{l}O_{n}\right]-E_{\mathrm{Vac}}^{*}\left[{\mathrm{Zn}}_{m-l}O_{n}\right]-lE_{\mathrm{Atom}}\left[M\right]$$

In this equation, *M* represents the element acting as a substitutional impurity in pure ZnO. $E_{\mathrm{Total}}\left[{\mathrm{Zn}}_{m-l}M_{l}O_{m}\right]$ corresponds to the total energy of the system, $E_{\mathrm{Vac}}^{*}\left[{\mathrm{Zn}}_{m-l}O_{m}\right]$ is the total energy of the system containing *l* Zn vacancies, and $E_{\mathrm{Atom}}\left[M\right]$ denotes the energy of an isolated atom of the element *M*. In what follows, we set m=n=16 and l=1. Furthermore, for M=Zn, we recover the formation energy of pristine ZnO.

**Figure 2:**
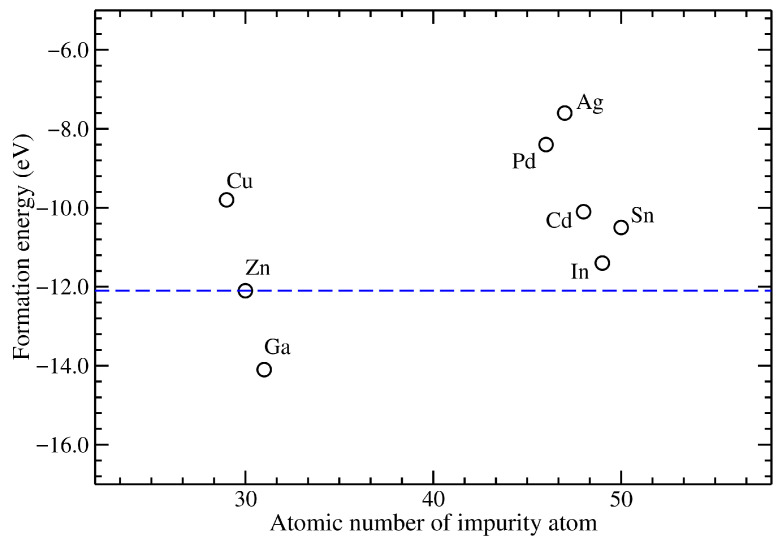
Formation energy as a function of the atomic number of the metallic impurity in ZnO. The horizontal dashed line corresponds to the pristine ZnO.

Figure 2 shows the formation energies per atom for all the systems under study. They are presented as a function of the atomic number of the metallic impurity, while a horizontal dashed line depicts the pristine case. Interestingly, Ga-doped ZnO exhibits lower formation energies than pristine ZnO, whereas In- and Sn-doped compounds have formation energies slightly higher than the undoped material. As formation energy is directly related with the experimental feasibility to synthesize the doped ZnO, it is interesting to note that In- and Sn-doped ZnO present the lowest formation energies. In contrast, Pd- and Ag-doped ZnO present the highest ones.

### 3.2. Electronic Density of States

Attending the low demagnetization energy barriers reported in Table 1, we concentrate on the paramagnetic regime of each system, except for the Cu-doped ZnO compound, which will be treated separately.

Since the Fermi level ($E_{Fermi}$) will serve as the reference energy in the subsequent diagrams, its accurate determination is crucial. To achieve this, the density of states curve is integrated until the number of electrons obtained matches the total number of electrons in the cell. The highest energy reached during this integration process corresponds to the informed $E_{Fermi}$ for each system.

The densities of states (DOS) for doped ZnO are presented in the following figures. In each case, the DOS of pristine ZnO corresponds to the grey filled curve, setting to zero the Fermi level of this system. Vertical dashed lines are added to locate the Fermi level of each doped system.

**Figure 3:**
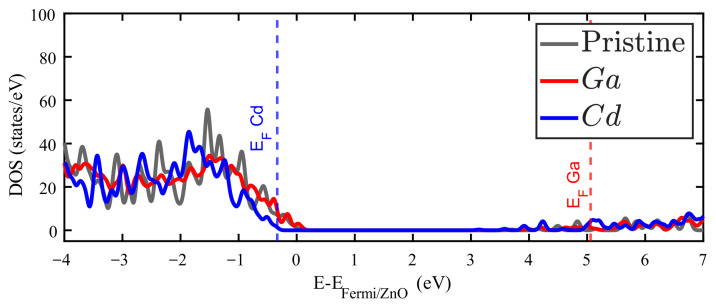
Density of states (DOS) for Zn_15_GaO_16_ (red filled line) and for Zn_15_CdO_16_ (blue filled line). Grey filled curve corresponds to the DOS for pristine ZnO.

As expected, doped ZnO exhibits a DOS similar to the pristine crystal. This is particularly evident below the Fermi level, because the substitutional metallic impurities considered here are all in the neighborhood of Zn in the periodic table. For instance, as Cd and Zn belong to the same group in the periodic table, the Cd-doped ZnO presents a DOS quite similar to the pristine crystal (see Figure 3). However, since the Cd atom presents an effective atomic number larger than Zn (acting as an acceptor impurity), the Fermi energy of this compound appears slightly below zero. This reflects the fact that most of the Cd valence electrons are tightly bound to this impurity. In the case of Ga-doped ZnO, the DOS remains quite the same as that of the pristine crystal, but the Fermi level is now at approximately 5 eV. Such dramatic change comes from the additional 4p electron provided by the Ga impurity, which finds the first empty states just above the original energy gap.

**Figure 4:**
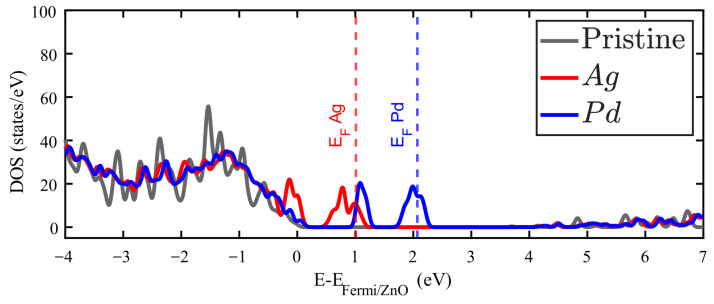
Density of states (DOS) of Zn_15_AgO_16_ (red filled line) and Zn_15_PdO_16_ (blue filled line). The DOS for pristine ZnO is plotted in grey.

**Figure 5:**
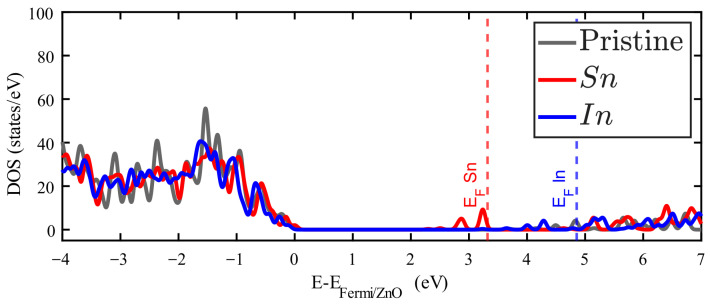
Density of states of Zn_15_InO_16_ (red filled line) and Zn_15_SnO_16_ (blue filled line). The DOS for pristine ZnO is plotted in grey.

Ag and Pd impurities introduce metallic electronic states just above the Fermi energy corresponding to the pure ZnO. Figures S3 and S4 of the Supplementary Materials show the band structure of these cases, and some bands crossing the Fermi level. The $4d^{10}$ orbitals of Pd and the $4d^{10}5s^{1}$ orbitals of Ag are responsible for the metallic behavior in these cases. This reflects that most of the Ag and Pd valence electrons are slightly bound to this impurity. On the other hand, In and Sn introduce occupied states around the first unoccupied states of pristine ZnO. It is in this zone where additional electrons from these impurities find available states. In any case, as Figure 4 and Figure 5 show that the general observation is that new occupied levels appear in the gap of the doped ZnO, which means a decrease in the bandgap as compared to the pristine crystal. The presence of these levels creates a new dynamics for these systems, which is important for the optical properties, particularly for low-energy photon absorption.

**Figure 6:**
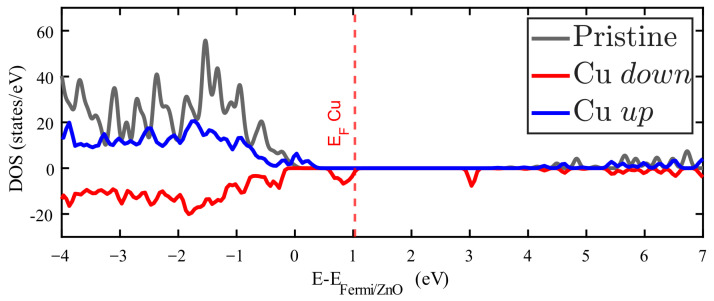
Density of states (DOS) of Zn_15_CuO_16_ splited according to its spin polarization: spin up (down) depicted in blue (red). The DOS for pristine ZnO is plotted in grey.

As stated before, only Cu impurities result in no null magnetic moment at room temperature. The unequal spin-polarized DOS just below the Femi level explains this feature, as is shown in Figure 6 (the pristine ZnO Fermi level is maintained at zero, while the red dashed vertical line shows the Fermi level for the Cu-doped ZnO). Furthermore, the new spin-polarized levels introduced in the forbidden energy band are interesting.

**Figure 7:**
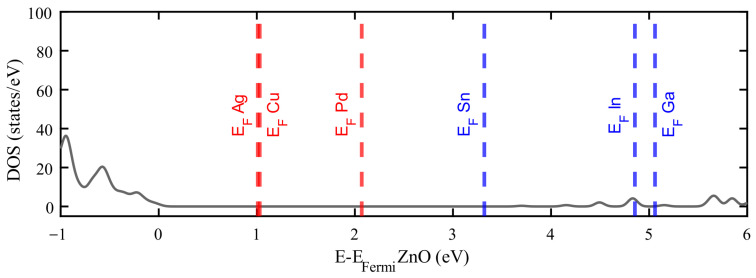
Comparison of Fermi level positions for various dopants relative to the pristine ZnO density of states (DOS—grey line). Dopants with fewer valence electrons than Zn are shown in red, while those with more are shown in blue.

It is possible to observe a systematic trend in the position of the Fermi level depending on the dopant employed. As shown in Figure 7, doping with Ag, Cu, and Pd results in an upward shift of the Fermi level up to 2.1 eV, while doping with Sn, In, and Ga produces a shift of 3.2 eV or more. This correlation between dopant and the Fermi level position suggests potential applicability in the design of *p–n* junctions.

### 3.3. Optical Absorption Properties

The linear optical response approximation was applied to obtain the electromagnetic absorption spectra. In this approximation, the imaginary part of the dielectric function $\varepsilon_{i}\left(\omega \right)$ is related to the optical absorption coefficient κ(ω) according to the following:

(2) $$\begin{array}{c} \kappa \left(\omega \right)=\frac{\omega \varepsilon_{i}\left(\omega \right)}{c\;n(\omega )}, \end{array}$$

where ω corresponds to the incident photon frequency, *c* is the speed of light, and *n* is the refractive index. In the framework of band theory without electron–hole interaction, $\varepsilon_{i}\left(\omega \right)$ can be obtained by applying the Drude–Lorentz equations [49,50,51]. Such a procedure is implemented in the *epsilon.x* toolset, which is available in the Quantum ESPRESSO [33] post-processing package.

**Figure 8:**
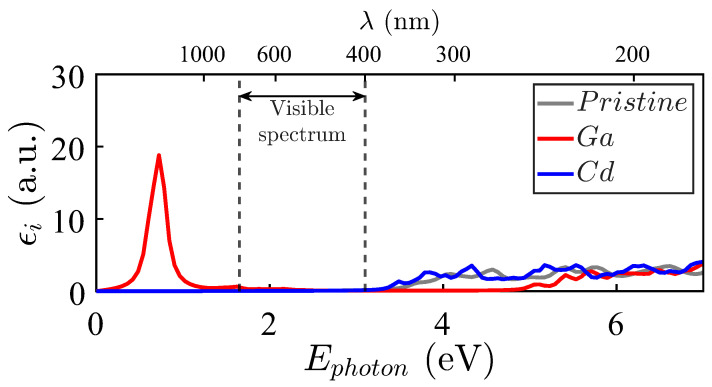
Imaginary part of the dielectric functions $\varepsilon_{i}\left(\omega \right)$ for Zn_15_MO_16_ against the pristine ZnO case (grey filled line). M = Ga (red filled line), M = Cd (blue filled line).

Figure 8 shows the imaginary part of the dielectric function $\varepsilon_{i}$ obtained for Zn_15_MO_16_: M = Ga, Cd. The imaginary part of the dielectric function for Ga (depicted in red) presents abundant bands displaced to lower energies. This result is explained by the couple of impurity levels just above $E_{Fermi}$ and the proximity of states in the valence bands (see Figure 3). In fact, the pronounced peaks at lower energies come from the perturbative terms containing the difference between valence and conduction energies in the denominator.

**Figure 9:**
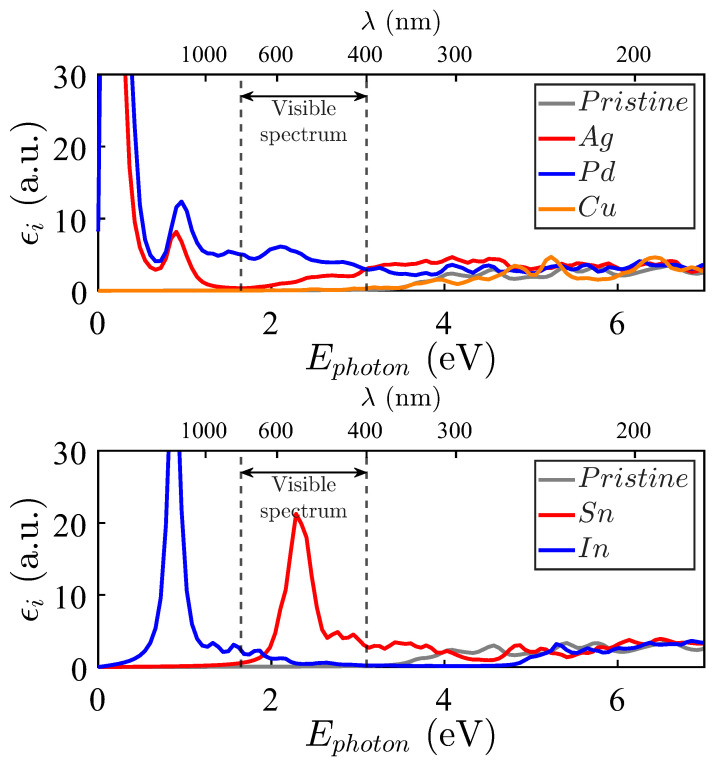
Imaginary part of the dielectric functions $\varepsilon_{i}\left(\omega \right)$ for Zn_15_MO_16_. **Upper panel**: M = Ag (red filled line), M = Pd (blue filled line), and M = Cu (orange filled line). **Bottom panel**: M = Sn (red filled line), M = In (blue filled line). The pristine ZnO case corresponds to the grey filled line.

The spectra of Cu, Pd, and Ag in Figure 9, upper panel, show that Ag and, particularly, Pd impurities activate the electromagnetic absorption in the visible spectrum, as well as in the near ultraviolet. ZnO doped with Pd and Ag shows pronounced peaks at lower energies, which also occur because of the proximity of the last occupied level and the first unoccupied one (see Figure 4).

While Cu-doped ZnO does not appear to exhibit electromagnetic absorption at the visible spectrum, it warrants separate analysis due to its non-zero magnetic moment at room temperature. In fact, the spin-polarized states at around 3 eV (see Figure 6) allow optical absorption in the visible spectrum (see Figure S5 of the Supplementary Materials). However, due to the spin conservation of the excited electrons, the imaginary part of the dielectric function results in much lower values than those in the cases of Pd- and Ag-doped ZnO.

The imaginary parts of the dielectric functions for the In and Sn systems are presented in Figure 9, lower panel. They present two leading structures at roughly 0.9 eV and 2.5 eV, respectively. The In-doped ZnO compound presents infrared absorptions because of impurity levels both below and above $E_{Fermi}$, while scarce activity is observed in the region of interest. However, the absorption peak induced by Sn impurities appears in the middle of the visible spectrum. In contrast, the near-ultraviolet spectrum is also notoriously active, while the absorption at lower energies is relatively low. In fact, these properties are very attractive for solar cell applications on the Earth’s surface and in outer space.

As can be appreciated, some substitutional impurities improve the electromagnetic absorption of pristine ZnO (whose $\varepsilon_{i}$ is depicted in grey color). Considering possible applications in photovoltaic technologies, the improvement in electromagnetic absorption induced by Pd and Ag impurities in the visible spectrum (between two vertical lines) and the near-ultraviolet transition regions is particularly interesting. No such optical absorption improvement is observed for Cd and Cu, which render an absorption spectrum quite similar to that of the pristine crystal, particularly in the visible and near-ultraviolet spectra.

### 3.4. Orbital-Resolved Density

**Figure 10:**
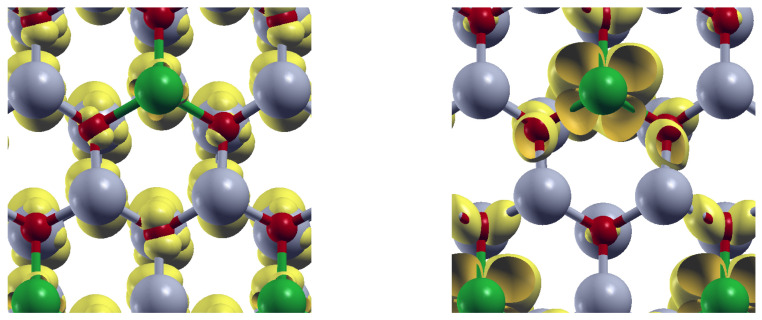
Orbital-resolved density (yellow) for Pd-doped ZnO at the valence band maximun (**left**), and at the conduction band minimun (**right**). Zn (O) atoms appear depicted in gray (red). Pd atoms are depicted in green.

To emphasize the role of the doping centers, it is illustrative to visualize the orbital-resolved density at the valence band maximum (VBM) and at the conduction band minimum (CBM). Figure 10 displays the orbital-resolved density (yellow) for Pd-doped ZnO, where Pd atoms are shown in green. The left panel reveals that at the VBM, the orbital charge density is primarily around O atoms (red). Similarly, the right panel shows that at the CBM, orbital charge density appears mainly around Pd atoms. Gray spheres represent Zn atoms.

**Figure 11:**
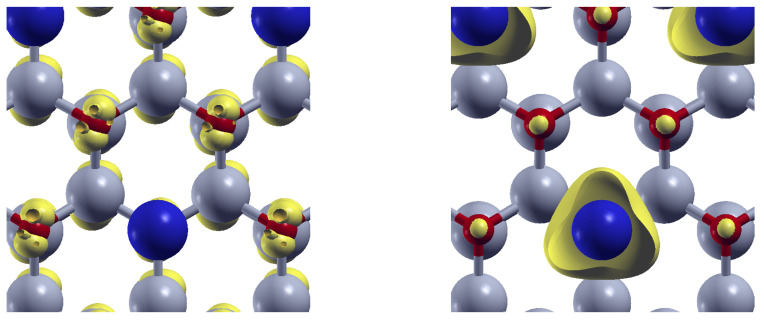
Orbital-resolved density (yellow) for Sn-doped ZnO at the valence band maximun (**left**), and at the Conduction band minimun (**right**). Zn (O) atoms appear depicted in gray (red). Sn atoms are depicted in blue.

The same situation is observed for Sn-doped ZnO. As depicted in the left panel of Figure 11, the orbital-resolved density is concentrated around O atoms at the VBM. However, at the CBM (right panel), the density shifts and localizes around the Sn atoms (blue).

In this way, the energy absorption induces charge transfer from O atoms to the doping centers, revealing the role of the substitutional impurities in the modifications of the optical absorption properties of the ZnO.

## 4. Conclusions

In this work, we conducted a comparative first-principles study of the structural, electronic, and optical properties of wurtzite ZnO doped with substitutional metallic impurities, specifically Cu, Ga, Cd, Pd, Ag, In, and Sn. Using DFT with Hubbard U corrections, we assessed how each dopant modifies the local electronic density of states and influences the optical absorption spectrum, particularly in the visible and near-ultraviolet regions.

Substituting Zn with metal elements in wurtzite ZnO introduces electronic states within the pristine bandgap. Each impurity modifies the density of states differently, creating diverse electronic pathways for potential applications. The modified energy gap shows considerable variation, ranging from nearly zero for Ag and Pd to wide gaps for Cu, In, and Sn, while the value for Cd remains similar to that of pure ZnO, as summarized in Table 1.

The absorption activity in the visible and near-ultraviolet regions is particularly relevant for solar energy harvesting. Among the tested impurities, Sn emerges as the most promising dopant for terrestrial solar cells due to its pronounced absorption in the visible range. This feature results in an advantage for multi-junction designs where narrow and intense absorption peaks can be exploited. Sn-doped ZnO also combines a favorable formation energy with additional absorption in the near-ultraviolet region (Figure 9).

Pd and Ag impurities enhance absorption across a broader spectral range, including the ultraviolet and infrared regions. This makes them ideal for space applications that require wide spectral coverage for single-junction cells. In contrast, Ga and In lead to narrow absorption peaks near 0.9 eV, which could be helpful for selective near-infrared sensors.

For the seven impurity metals considered, spin-polarized results show no significant deviation from non-magnetic calculations and can be disregarded for room-temperature applications. In the case of Cu, although it exhibits magnetization at room temperature, its optical properties are not of interest for applications in the visible or near-infrared spectrum.

Our results indicate general trends rather than exact values. Therefore, experimental validation and more sophisticated theoretical approaches—including variable dopant concentrations, temperature effects, and excitonic corrections—are essential to fully assess the applicability of these materials in optoelectronic and photovoltaic devices. Overall, this study provides a foundational comparative analysis that may guide future research into ZnO-based materials doped with earth-abundant metallic elements.

## Figures and Tables

**Table 1:** Lattice constants, magnetic moment (μ), demagnetization energy per atom ($\Delta \epsilon_{p}=\left(E_{paramagn}-E_{spin-polar}\right)/N$), and energy bandgap ($E_{g}$) for different substitutional metallic impurities on ZnO.

Impurity	a	b	c	c/a	μ	$\Delta \epsilon_{p}$	$E_{g}$
		**Å**			**μB/cell**	**meV**	**eV**
Cu	3.212	3.212	5.126	1.596	1.0	32.6	2.1
Ga	3.209	3.209	5.143	1.603	0.0	0.0	0.2
Cd	3.248	3.248	5.199	1.601	0.0	0.0	3.5
Pd	3.217	3.217	5.164	1.602	1.9	3.5	0.0
Ag	3.218	3.218	5.155	1.602	0.0	0.0	0.0
In	3.225	3.225	5.164	1.601	0.0	0.0	0.2
Sn	3.220	3.220	5.194	1.623	0.0	0.0	1.4
(Pristine)	3.206	3.206	5.126	1.599	0.0	0.0	3.4

## Data Availability

The original contributions presented in this study are included in the article. Further inquiries can be directed to the corresponding author.

## Supplementary Materials

The following supporting information can be downloaded at: https://www.mdpi.com/article/10.3390/ma18194613/s1, Figure S1: k-mesh convergence; Figure S2: band structure of pristine ZnO; Figure S3: band structure of Pd-Doped ZnO; Figure S4: band structure of Ag-doped ZnO; Figure S5: The imaginary part of the dielectric function of Cu doped ZnO.

## Abbreviations

The following abbreviations are used in this manuscript:

DFT	Density Functional Theory
DOS	Density of states
LDA	Local density approximation
QE	QuantumEspresso
