# Supplementary material for: Optical Absorption Properties of Sn- and Pd-doped ZnO: Comparative Analysis of Substitutional Metallic Impurities
Source: Materials (Basel). 2025 Oct 5;18(19):4613. doi: 10.3390/ma18194613 (PMC12526443; doi:10.3390/ma18194613)
Supplement: Supplementary file 1 [file materials-18-04613-s001.zip › materials-3846173-supplementary.pdf]

Supplementary Materials: Optical absorption properties of Sn-doped ZnO: a comparative analysis of substitutional metallic impurities

Vicente Cisternas[1], Pablo Díaz [1], Ulises Guevara[2], David Laroze [3] and Eduardo Cisternas[1,*]

1. k-mesh convergence

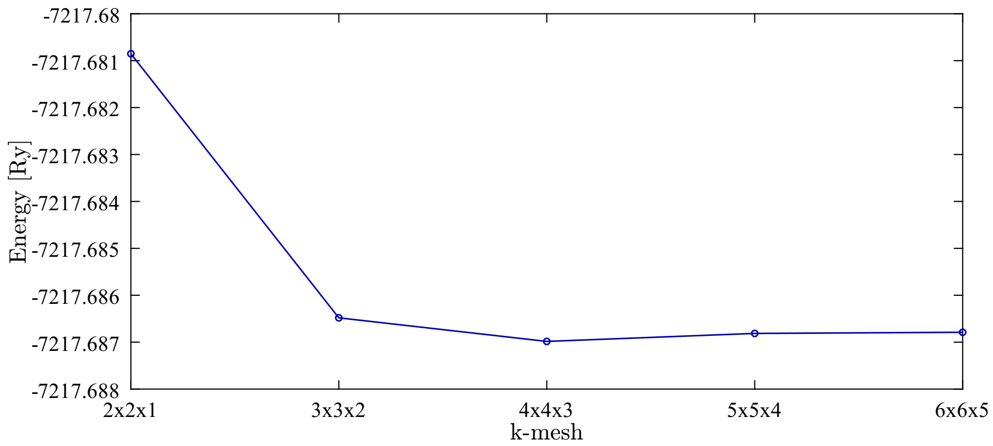

Figure S1. Total energy as a function of the k-mesh.

2. Band structure of pristine ZnO

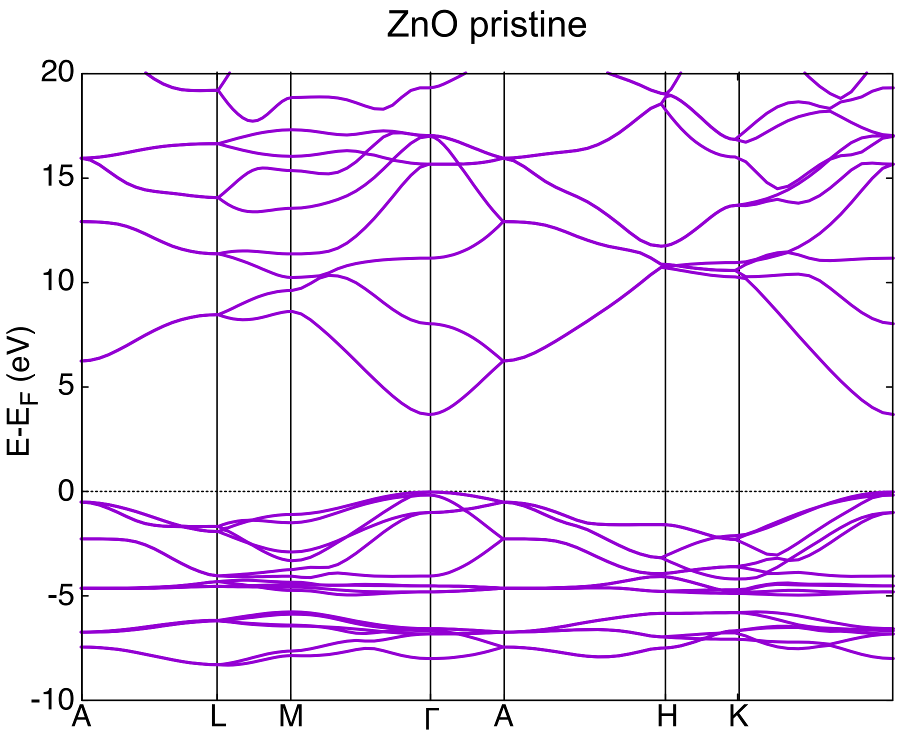

Figure S2. Band structure of pristine ZnO (wurzite phase).

3. Band structure of pristine of Pd- and Ag-doped ZnO

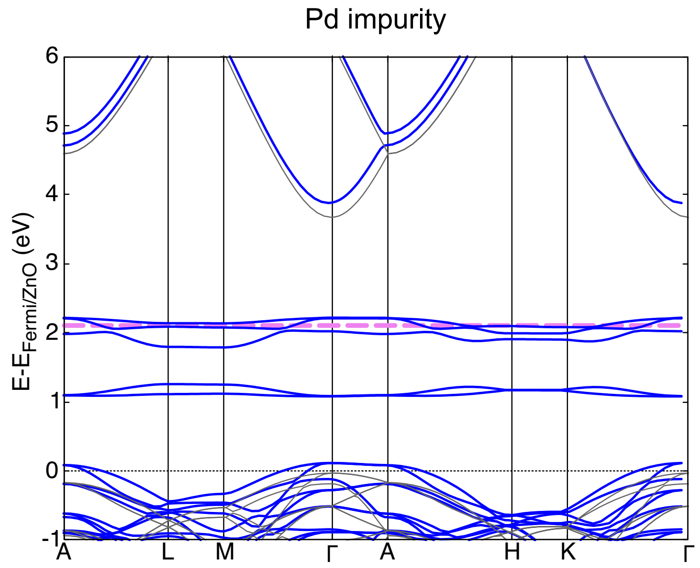

Figure S3. Band structure of Pd-doped ZnO. The horizontal dashed line (violet) shows the Fermi level of the doped system.

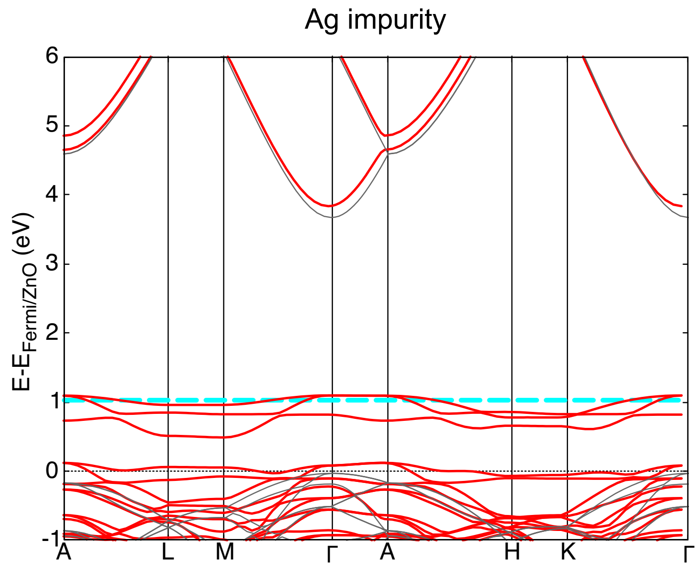

Figure S4. Band structure of Ag-doped ZnO. The horizontal dashed line (cyan) shows the Fermi level of the doped system.

4. Optical absorption for Cu-doped ZnO: magnetization effects

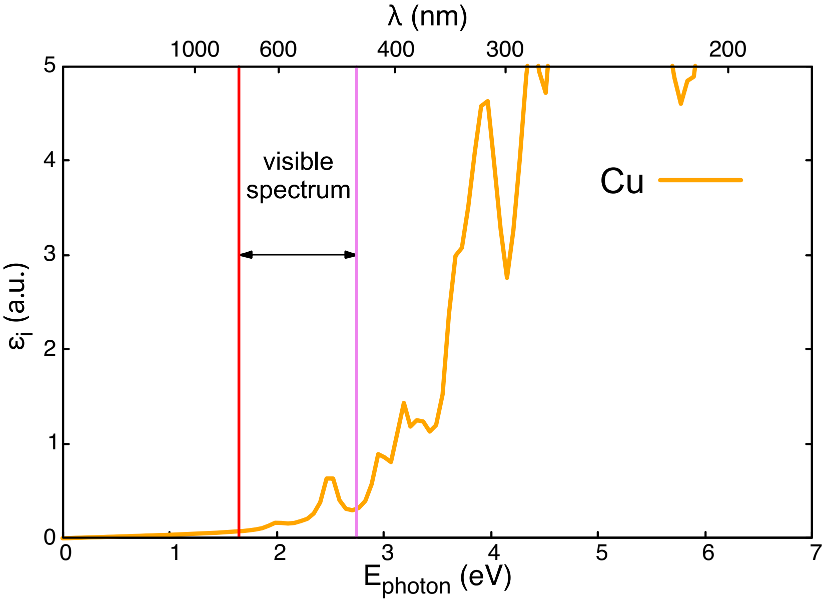

Figure S5. Imaginary part of the dielectric function $\varepsilon_i(\omega)$ for $Zn_{15}CuO_{16}$.
